# The impacts of Bolsa Família, Cisterns, and PRONAF on rural women’s food practices in Northeast Brazil: a qualitative analysis

**DOI:** 10.1186/s12939-025-02653-6

**Published:** 2025-10-15

**Authors:** Mariana Lopes Simões

**Affiliations:** School of Public Health - Graduate School “Health Policy and Systems in Uncertainties” (GRASP)/ AG3 - Epidemiologie & International Public Health, Bielefeld University, Universitätsstraße 25, 33615 Bielefeld, Germany

**Keywords:** Nutrition, Gender equality, Public policies, Agriculture, Food practices

## Abstract

**Background:**

Brazilian Zero Hunger strategy included the cash-transfer program Bolsa Família, the credit to strengthen family farming PRONAF, and providing cisterns. Understanding the effects of this strategy in rural areas and pondering gender is crucial as women play an essential role in family’s dietary decisions and agriculture. This study explored perceptions of women from a rural community in Northeast Brazil regarding the impact of the three programs mentioned on their food practices.

**Methods:**

A qualitative study was conducted in a rural setting with severe drought. Seventeen women, 18 to 87 years old, were selected through convenience sampling and interviewed in-depth. Data were analyzed using thematic analysis.

**Results:**

This study examines rural women’s perceptions of three Brazilian social programs (Bolsa Família, Cisterns, PRONAF) across food production, distribution, preparation, consumption, and waste management. The cistern program significantly impacted food production and preparation by saving time, improving water access, and enabling diversified farming. PRONAF allowed some families to purchase livestock, but with traditional gender roles persisting, credit decisions remained male-dominated. Bolsa Família impacted food preparation and consumption by enabling access and increasing dietary variety but did not necessarily enhance nutritional quality. Key limitations of the programs regarding food practices include: 1 failure to address structural gender inequalities in agriculture that could allow an active role for women in agriculture and support distribution of production; 2 insufficient measures to counter rising processed food consumption; and 3 waste management systems that lag behind increased consumption, leading to practices harmful to both health and the environment. The cistern program stands out by addressing multiple needs (water, time, agriculture) without disrupting local practices.

**Conclusion:**

Interviewees have more access to and diversity of food. Their diet is still composed of no or minimally processed food, but an increase in the consumption of ultra-processed food was observed. Their power of decision is restricted to the domestic sphere, with little participation in using PRONAF. Transformative change requires gender-sensitive program redesigns—such as empowering women in credit decisions—and better integration of waste management and nutrition education. Without these reforms, programs risk improving food access while leaving systemic inequities unresolved.

## Background

Women play an essential role in their families’ dietary decisions and in agricultural work worldwide. In rural settings, evidence shows that stimulating their autonomy in production positively influences maternal and child dietary diversity and nutritional outcomes [1, 2]. Furthermore, women’s contributions to agriculture are crucial for advancing both high-tech and traditional agricultural sectors toward greater economic, social, and environmental development [3].

Brazil’s population is predominantly urban, with only 13.4% in rural areas [4]. Nearly half of the rural population is concentrated in Northeast of the country, where women are more likely to manage rural properties compared to other regions [5]. This region is partially covered by the Caatinga, a semiarid biome characterized by an erratic rainfall distribution. Frequent droughts in the area disrupt subsistence agriculture, affecting the diet of local residents in terms of food access, quantity, variety, and quality [6, 7], increasing food insecurity rates and worsening socioeconomic conditions [8].

With this, the Northeast falls behind in terms of human development indicators (HDIs). In 2020, it recorded the second-highest underfive mortality rate among Brazil’s regions, with 14.8 deaths per 1,000 live births, exceeded only by the North. In comparison, the Southeast, South, and Central-West regions reported lower rates of 11.9, 10.4, and 12.9, respectively [9].

In 2003, Brazil launched a comprehensive strategy to eradicate hunger, comprising a range of programmes to guarantee access to food, strengthening family farming, income generation, and social mobilization and oversight [10]. This initiative aimed to cover various dimensions of the Sustainable Development Goals (SDGs), including rural livelihoods, food security, health, agricultural production, and land-use change [11].

Three programmes of this strategy have directly impacted rural women from the northeast region: the Bolsa Família Programme, a conditional cash transfer programme implemented in 2004 that provides financial aid to vulnerable families, with a focus on education, health, and social inclusion, prioritizing women as primary beneficiaries [12, 13]; the Cistern Programme, initiated in 2003 through a government‒civil society partnership, which addresses water scarcity in Brazil’s semiarid region by enabling families to harvest and store rainwater through low-cost cisterns, ensuring water access for consumption and agriculture [6]; and the National Programme to Strengthen Family Agriculture (PRONAF), which has existed since 1995 but was incorporated into the strategy to support small-scale farmers with subsidized credit to increase production, reduce rural poverty [14], and promote gender-inclusive financing by creating specific credit lines for women [15].

Food emerges as a core organizing principle in daily life, mainly led by women, and can be a nexus for conceptualizing sustainable changes in public policies [16]. Given the important role women play in agriculture, evaluating the relationships of these three Brazilian programmes with women’s food practices is crucial. However, the country’s variety of cultural practices, beliefs, and traditions complicates the process of gathering evidence on the effectiveness of a strategy, particularly when factors such as gender and race are considered, given that these variations can lead to differences in how people experience and respond to policies and strategies. Additionally, logistical difficulties further complicate the ability of researchers to reach dispersed rural populations located in Northeast Brazil [17].

This study aims to analyze the perceptions of women from a rural community in Paraíba, Northeast Brazil, of the programmes Bolsa Família, Cisterns, and PRONAF—of their food practices. Given the specificities of the rural areas, for the purpose of this study, food practices are defined as the dimensions related to the act of eating, which include the production of food (agricultural crops and livestock farming), distribution (the destination of the production), meal preparation (including acquisition of food), meal consumption, and handling leftovers.

## Methods

### Research design

A qualitative study design was chosen to analyze the programmes, as it provides an understanding of the context, beneficiary perspectives, and policy outcomes [18]. In research on food practices, qualitative methods help bridge the gap between quantitative data and the complex roles that food plays in participants’ lives by exploring their perceptions and contextual influences on dietary habits and health behaviors [19, 20].

### Sampling

The target population of this study was women from the rural community of Balanço, which belongs to the municipality of Sumé and is located in the state of Paraíba, Brazil. The criteria for participation included being above 18 years of age—the age of majority in Brazil—and being physically and mentally capable of completing the interview independently. The sample strategy used was convenience sampling.

### Setting

Sumé has a population of 17,166 people within an area of 833.315 km², according to the Brazilian Census of 2022. The urbanized area of the district (characterized by the presence of culverts, sidewalks, paving, and curbs) is 3.63 km², constituting 0.44% of its total territory. The percentage of basic sanitation coverage is 24.2% [21].

In terms of population distribution, 76.18% of Sumé’s inhabitants live in urban areas, whereas 23.82% reside in rural regions [21]. The infant mortality rate in Sumé was 17.94 per 1,000 live births in 2021, which was higher than the rate for Paraíba, which was 12.63 in the same year [22]. The HDI for Sumé was 0.627 in 2010 [21].

In 2022, 11.26% of Sumé’s population was employed in the formal labor market, earning an average of 1.7 times the minimum wage (approximately 292.18 euros). Additionally, 47.6% of the population received half of the minimum wage (approximately 115 euros) monthly from other sources, such as the cash transfer programme Bolsa Família [21].

The rural community of Balanço belongs to Sumé and is located 24 km from the town, at coordinates − 7.498749, -36.939663. It consists of 32 houses situated along 1.3 km of the main road. The houses are not connected to a sewage system, and 30% of them are equipped with cesspits [23]. The village does not have a public waste collection system.

The primary livelihood activities of Balanço’s residents are subsistence farming, including crops of beans, corn, and vegetables, such as potato, pumpkin and cassava, as well as animal rearing, particularly cattle, goats, sheep and poultry.

### Ethical approval

This study was submitted to the Comissão Nacional de Ética em Pesquisa do Conselho Nacional de Saúde (National Committee for ethical in research – National Health Council) through the process #61674322.0.0000.0209 and was approved under the number 5.706.890, in accordance with the Resolution 510/2016 from the Brazilian National Health Council.

### Procedure

The data were collected through individual semistructured interviews, aiming to capture the perceptions of the rural community’s interviewees regarding the relationship between their food practices and the programmes studied. An interview guide and a consent form were prepared in advance of the interviews. The guide consists of two sections: the first section features closed-ended questions designed to gather pertinent socioeconomic and demographic information of the participants, and the second section includes open-ended questions addressing the targeted social programmes and the participants’ food practices. Examples of the questions can be found in Table 1 – Examples of the interview questions. The consent form was developed in Portuguese, the native language of both the interviewees and the interviewer, according to the standards of Brazilian resolution CNS n° 510 of 2016.

**Table 1:** Examples of the interview questions

1	Please describe your favorite foods and regional food traditions.
2	Have your agricultural productions changed over the past two decades? If so, what new crops/livestock have you adopted, and which have you discontinued?
3	What technological or varietal changes have you implemented in your farming since the early 2000s?
4	How have Bolsa Família, Cisterns, or PRONAF influenced your household’s food production, distribution, preparation, consumption or the management of left overs?
5	Thinking back, are there foods you once ate regularly but do not eat anymore? What were they? What led you to stop eating those foods?
6	How would you describe your current ways of obtaining food? Do you believe this is significantly different from how you accessed food years ago? (If yes) "Could you explain these differences and how you feel about them?

The fieldwork for this research was conducted in October 2022. Among the 32 houses in the village, 22 had at least one resident who met the criteria to participate in the study. Residents from 15 houses agreed to participate. In two of these houses, two women each were eligible to participate, and in both cases, they were interviewed.

In one of the houses, three women were initially classified as eligible to participate in the research. However, during the interview, one of them admitted to being 17 years old, and another, owing to a mental condition, was unable to answer the questionnaire independently and required support from other member families. These interviews did not meet the inclusion criteria for participants and were therefore discarded.

In total, 19 women were interviewed, and 17 interviews were considered for the data analysis. The conversations were conducted in Portuguese and began with an introduction to the research and its objectives, followed by the signing of the consent form.

Each interview lasted between 30 and 60 min and was audio-recorded. The interviews took place in the morning, and the audio was transcribed later the same day in the afternoon or on the weekend following the interview via MAXQDA Plus 2022 Network software. Afterward, notes and memos taken during the fieldwork were added to MAXQDA software.

The data were assessed via thematic analysis. This method is well suited for understanding common experiences, thoughts, or behaviors across a dataset, as it enables the identification of recurrent patterns [24, 25]. The process involved creating themes that emerged from identifying patterns describing the phenomenon within the data, allowing for both description and interpretation of the data’s meaning [26, 27].

To build the themes, the data were analyzed from the bottom up in an inductive process. This began with the examination of the interviews, memos, and notes taken during the fieldwork, searching for patterns from the raw data, followed by the creation of codes that generated the themes. Next, the data were reviewed deductively, moving from the themes back to the raw data, to determine whether more evidence could support each theme or if additional information needed to be gathered [28].

## Results

This study analyzes findings from 17 interviews with women residing in the Balanço community. The median age of the participants was 54 years (ranging from 18 to 87). One participant declared herself white, four as Black and twelve as mixed race (referred to as *“Parda”* in Portuguese). Participants had between 0 and over 10 children, with median of 3 and the most common number being 1.

With respect to programme coverage, the Cistern Programme has the greatest number of beneficiaries, with 13 women directly benefiting and 3 benefiting indirectly, meaning that they lived in a household where someone else had previously received support from the programme.

The Bolsa Família cash transfer programme is directly received by 9 women. The other 3 women benefited indirectly, as their mothers had received payments during their childhood.

The PRONAF programme was used by five participants in this study.

Fifteen out of seventeen participants spent their lives in rural areas between the states of Paraíba and Pernambuco. They continue the work of previous generations in family agriculture, which remains the primary economic activity in the region.

Table 2, titled “Participants’ socioeconomic overview,” summarizes the general socioeconomic situation of the participants.

**Table 2:** Participants’ socioeconomic overview

	Color	Primarily Rural Residence	Marital Status	Engaged in Agriculture?	Education Level	Main Income	Formal Employment Status
1	*Parda*	Yes	Unmarried Partner	Yes	High school	No income	Student
2	White	Yes	Married	Yes	Higher education	Trainee	Trainee - Teacher
3	*Parda*	Yes	Single	Yes	Technical school	Formal work	Employed – Community Health Worker
4	*Parda*	No	Divorced	Yes	Elementary school	Bolsa Família	Freelancer - Cleaner
5	*Parda*	Yes	Married	Yes	No formal education	Bolsa Família	Unemployed
6	*Parda*	Yes	Unmarried Partner	No	Elementary school	No income	Unemployed
7	*Parda*	Yes	Single	Yes	High school	Bolsa Família	Freelancer - Cleaner
8	*Parda*	Yes	Married	Yes	Elementary school	Bolsa Família	Unemployed
9	*Parda*	Yes	Unmarried Partner	Yes	No formal education	Bolsa Família	Unemployed
10	*Parda*	Yes	Married	Yes	No formal education	Pension	Unemployed
11	Black	Yes	Single	Yes	No formal education	Retired	Retired
12	*Parda*	Yes	Married	Yes	No formal education	Retired	Retired
13	Black	No	Single	Yes	Technical school	Retired	Retired
14	*Parda*	Yes	Married	No	No formal education	Retired	Retired
15	Black	Yes	Single	No	No formal education	Retired	Freelancer - Artisan
16	*Parda*	Yes	Widow	Yes	No formal education	Retired	Retired
17	Black	Yes	Widow	No	No formal education	Retired	Retired

Note: The Bolsa Família programme was replaced by the Auxílio Brasil programme on November 20, 2021. However, the programme’s content remained largely unchanged, with the main difference being the name and certain administrative adjustments. The programme was renamed Bolsa Família again on March 1, 2023, with no significant alterations to its structure or benefits

Table 3 summarises the reported effects of the Bolsa Família, PRONAF, and Cisterns programmes on the five dimensions of food practices examined in this study: production, distribution, preparation of meals, consumption, and handling of leftovers. The table reflects only the effects described by participants during interviews, without interpretation or value judgement, and illustrates the distinct ways in which each programme influenced different aspects of household food practices in the rural community.

Building on these findings, this study identifies four key themes that capture the participants’ experiences and perspectives. These themes are detailed as follows:

**Table 3:** Description of the effects of three social programmes on women’s food practices in rural paraíba

Dimension	Bolsa Família (BF)	PRONAF	Cisterns
Production	Used by one participant to buy seeds; otherwise, no direct effect reported.	Enabled purchase and sale of larger animals; construction of cisterns.	Provided water for cultivating gardens and raising animals.
Distribution	No direct effect reported.	Enabled sales of larger animals acquired through the programme.	No direct effect reported.
Preparation	Increased variety; facilitated access to vegetables, meat, fruits; replaced home-produced carbohydrates with purchased ones.	Increased availability of animal products from livestock.	Provided potable water for cooking; facilitated cleaning of produce.
Consumption	Increased consumption of vegetables, meat, fruits, and ultra-processed products, with the introduction of new dishes while preserving traditional meals.	Increased consumption of animal products from livestock.	Increased consumption of fresh produce from gardens.
Handling leftovers / waste	Increased combustible and non-combustible waste; combustible waste burned; non-combustibles stored.	No direct effect reported.	No direct effect reported.

### Food practices in the caatinga: changes and remains

The first theme emerging from the interviews highlights how women in the Balanço community describe the characteristics that have changed and those that have persisted in their food practices in the past 20 years (considering that the implementation of the programmes occurred between 2003 and 2004) without establishing a direct link between these characteristics and the programmes.

With respect to food production, they keep the cultivation of beans, corn, and cassava, along with chives and cilantro, in small gardens. Over the past two decades, an increased variety of vegetables and fruits have been reported. Their gardens now include bell peppers, lettuce, eggplants, cucumbers, and herbs. Traditional fruit trees such as papaya, watermelon, guava, cashew, embu, mango, and sugar-apple remain, with new additions such as soursop, lemon, orange, acerola, and pomegranate.

The main change in animal husbandry is the number of animals raised, not the variety or technique. Fourteen women raised poultry in their gardens, primarily chickens and some turkeys. Only two of them raise goats with their husbands’ help, and one buys cattle to fat and sell, a task handled by her husband or son.

According to the participants, the food distribution has not changed. The habit of donating, within the community, of surplus production is maintained for most of these women:

*I do not sell anything… one son comes and takes it*,* another one comes and takes it*,* the neighbor comes and takes it*,* another one comes and takes it. P.13.*

Three women reported occasionally selling their products. One sold chickens once to pay an electricity bill, whereas two sold small animals or herbs when buyers appeared.

With respect to acquisition and preparation, these women noted an increased expenditure on supermarkets by buying foods and new, previously inaccessible items. Most of their food now comes from the market. Except for the two youngest participants, all other interviewees remember a time when they relied mainly on their production to have food but could not pinpoint when this changed. They attributed their past self-sufficiency to financial constraints limiting access to city markets and supermarkets.

*I remember because at the minimarket we used to buy on the cuff… we would go there and the man would sell it to us*,* we would come*,* then when we had a day’s work cleaning out other’s crops*,* we would get that money*,* go there and pay …. today we have enough money*,* we go to the city and buy there. P.10.*

The programmes have also impacted the consumption patterns. The interviewees reported adding minimally processed foods such as rice, pasta, and vegetables to their diets. They frequently cited the introduction of meat as the main dietary improvement after the programmes, and less often, the addition of vegetables and fruits:

*I added mainly the meat*,* that we could not afford to buy the meat… P.4*

*Today*,* you have the best meat…. to buy a piece of meat it used to take the hardest work in the world …. P.8.*

One participant credited the increase in the consumption of vegetables to the increase in her knowledge regarding healthy food:

*Yes*,* I eat them (the vegetables) more now… because it is like this*,* we are getting older*,* and we are becoming more aware of… the power of food*,* right…. P.4.*

The participants described the shift from traditionally prepared regional foods to ready-made supermarket options. For example, cassava starch and corn couscous, once made locally or at home, are now purchased premade.

All the participants mentioned rice and noodles as new carbohydrate staples added to their meals, supplementing beans, meat, and vegetables. Some local dishes were withdrawn from their menu. *Xerém*, a cornmeal, and *Biju*, a traditional manioc cake, are considered foods that are no longer part of daily meals because of the workload to prepare them. One participant no longer ate xerém because it reminded her of the times of scarcity. The two younger participants reported a different food-related activity: going into the city to eat pizza with friends.

Despite having refrigerators, the community’s women continue to cook daily, as they dislike the taste of food stored for more than a day. They still primarily use coal for cooking; however, all of them have been able to buy a gas stove in the past 20 years, which they also use, although less frequently. Rice and beans remain the favorites of most women, with the exception of the youngest interviewee, who prefers pasta and meat.

The primary change in how leftovers are managed is that grocery packaging is burned, due to the absence of public garbage collection in the area.

### From field to plate: insights into the development of labor among women from the rural community Balanço

This theme explores the programmes’ impact on the work of rural women in the Balanço community. Their work remains primarily domestic and agricultural, covering all activities toward food practices, with some holding paid jobs outside the home (see Table 2—Participant’s socioeconomic overview). All interviewed women handle housework, including maintaining vegetable gardens and raising poultry. Among those not currently engaged in agricultural fieldwork, two are students, one helps her husband run a mini-market, one has health issues, and the two oldest women are unable to work due to age. These last four did fieldwork when they were younger. All participants over 30 years of age recalled having to leave school to work in the fields or balance both.

*When I was still in school*,* I used to go to school in the morning*,* and in the afternoon*,* I’d get back in time to go to the fields and pick beans; I’d help my father pick beans. P.08.*

There is no perception of change in the work developed in the field. The women interviewed affirm that they participate in the phases of land preparation, cleaning the fields (owing the plants), planting the seeds, postplanting maintenance, and harvesting. Partners and/or children and relatives are often involved in the fieldwork with them. Only one participant who lived alone and maintained a field claimed to pay helpers to perform tasks that she could not perform.

The women interviewed understood that the work in the field has been decreasing, and they attributed this to the reduction in rainfall, the impossibility of heavy work due to their age, and the lack of involvement of the younger generations in supporting the work:

*I can see it is finishing*,* the agriculture culture… it is not because… because if you take a boy from school to work in the fields*,* they will call you. P.08–44.*

Except for the two youngest interviewees, all interviewees noted that the main change in their work since the implementation of these programmes was related to water collection, which impacted food production and preparation. Before the cisterns were installed, they had to carry 20 L of nonpotable water on their heads for 1–3 km and then boil it before use. Many participants mentioned this task.

*I could feel my head burning*,* everything felt like burning*,* the can was hot. P.10.*

The quality of the water collected was also described multiple times:

*The Guará Wolf would bathe in the water*,* and we would feel the taste of it in the water*,* the smell…. It had that taste*,* but we need to bring it and use it to shower*,* do the dishes*,* do everything. P.08.*

Other tasks that remained the responsibility of the women interviewed were household shopping and food preparation. Five participants mentioned passing down this responsibility from mother to daughter.

In only one case, a participant who does the grocery shopping for her mother’s family also lives with them. In other cases, participants buy food for both their own household and their mother’s household.

Finally, these women also manage the organic waste, separating it to give it to the animals. In contrast, the work of burning other kinds of waste is divided among the various residents of the house.

### Views on the impact of the target programmes on the participants’ food practices

This theme explores the direct mentions of the programmes made by the interviewees.

The cistern programme is associated with access to clean water. The youngest interviewee was the only one who did not know about the existence of this programme. However, she was aware that a cistern was available in her house. The programme was also often connected with the reduction of work carrying water, and the availability of water at home was remembered as a means to facilitate work in preparing food by allowing the use of clean water for drinking, cooking, and washing food, as well as the planting of small vegetable gardens, which also impacts food production:

*We used to carry the water in cans in our heads*,* so it would be just the necessary for the basic things. Currently*,* we have water from the cistern*,* so we wash vegetables. P.10.*

All the interviewees were aware of the BF programme and associated it with improved purchasing power. The money they received allowed them to buy different types of food, hygiene products, and snacks for their children at school, although it was also used to buy hygiene items, gas, and electricity. Two of the women interviewed reported using money from the programme to buy seeds for planting.

The BF programme was also remembered for not keeping up with inflation prices in Brazil. Some interviewees reported that they no longer buy beef and gas because of the current prices:

*In the beginning*,* you were able to buy*,* for example*,* two kilos of meat or three or four for a house with many people; currently*,* you cannot buy anymore. P.12–66.*

Two interviewees had never heard of the PRONAF, and ten were aware but had never used it. Of the five women who used the PRONAF, two bought animals, two built cisterns, and one built a water reservoir for raising animals. The decision on how to use the funds always involved a familiar male member, for instance, the husband or father.

All the interviewees agreed that the programmes have generally reduced hunger in the region. Some expressed concerns about the potential loss of these benefits:

*If… if today there’s a programme… tomorrow there’s a sift through the beneficiaries… some people have already been removed*,* so you cannot rely 100% on a government programme. P.4.*

### A view of rural women regarding the relationships among food, health and the environment

The fourth theme from the interviews emerged from spontaneous comments about diet, environment, and health. Although not directly asked, the participants frequently mentioned the connection and shared their opinions. A common topic was the quality of the food available to buy:

*Before you would have a healthy chicken*,* a healthy goat*,* a healthy beef*,* and today you do not have it because everything comes with so many vaccines*,* so many things*,* so many diseases*,* so many pesticides… P.03.*

Although they emphasized the main change in their food consumption as being the introduction of meat and minimally processed food, the mention of the acquisition and/or consumption of ultra-processed foods was made by all participants:

*I got there*,* at the supermarket*,* and saw those little things*,* and I thought that there was a spice that people use*,* the Knorr©*,* which was to make soup… or some meat*,* something like that… so I did that. I truly liked it… Knorr©…I even liked to put a little in the beans…to make the beans tastier. P.11.*

The participants were often aware of the effects of the ingestion of ultra-processed food on health:

*I used to like it a lot… after I watched the news and saw a report about the mix of meat it (sausages) has… I stopped buying those things. P.11.*

Regarding their habits while having a meal, most participants set the table and eat with their families. However, four now usually eat alone on the sofa while watching TV, with one acknowledging this as an unhealthy practice. Three women with school-aged children mentioned difficulty getting their kids to join family meals:

*When I manage to sit at the table with my mother and my son*,* it is because I’m there nagging him about the importance of it P.03.*

Several participants talked about the sensation that the rains in the region have been decreasing lately:

*I sometimes think like*,* is the time getting short? I think like this: is the world coming to an end? Because we’re seeing everything like this*,* it is different*,* the rains are few… the fruits are few… it is not like it used to be. P. 10.*

All the participants agreed that their garbage production has increased over the years. They were advised by the public system to burn waste to prevent disease-causing animals and farm animals from eating plastic. One woman reported health issues from burning waste:

*When I’m going to set the garbage on fire*,* I put it on and leave immediately because otherwise*,* I will suffocate with the smell of smoke. Even when the neighbors are setting garbage on fire there*,* I leave the house because I cannot stand it. My nose starts to burn; my eyes start to cry*,* and it gives me a strong headache. P.01.*

Six women—the youngest ones—agree that it would be better if the region would have waste collection:

*If a truck would come by, even if it is once a week, picking it up… because that is how it is, there’s the glass, we can’t throw it anywhere, it is separated, so there’s the glass and the aluminum too, like the can… I think that would be very good, because the other things we burn right, but especially glass, it is a very dangerous thing. P. 04.*

Two of the women participating in this study believe that the current system where they burn their waste well meets the demands of the region and does not need any changes; the others do not have an opinion about it.

## Discussion

This study aimed to analyse the perceptions of women in a rural community in Paraíba, Brazil, of the impact of three social programmes, Bolsa Familia, Cisterns, and PRONAF, on their food practices.

The findings reveal that while BF, Cisterns, and PRONAF have meaningfully impacted food production, preparation, consumption, and leftover management in this community, their transformative potential remains constrained by structural barriers. Three key limitations emerge: [1] programmes fail to address deeply rooted sexist disparities in agricultural decision-making (particularly credit use via the PRONAF) [2], nutritional education components remain inadequate to counterbalance the trend toward ultraprocessed food consumption, and [3] waste management systems have not evolved to match rising consumption patterns, creating new environmental health risks. These limitations reflect broader challenges in Brazil’s intersectoral policy implementation. The Brazilian National Food and Nutrition Policy (Política Nacional de Alimentação e Nutrição - PNAN) [29] is grounded in the rights to health and to food, guided by the doctrinal and organizational principles of the Unified Health System (SUS), and complemented by commitments to cultural diversity, autonomy, social determinants, interdisciplinarity, and food and nutrition security with sovereignty. Yet, as Alves and Jaime [30] argue, these principles are often undermined in practice by fragmented implementation and insufficient coordination across sectors.

To address these gaps and enhance the transformative potential of existing programmes, Table 4 synthesizes public policy recommendations grounded in this study’s findings:

**Table 4:** Policy recommendation

Policy Area	Recommendation	Purpose / Expected Impact
Women’s Empowerment	Simplify credit application procedures for women; provide targeted financial literacy and technical assistance.	Increase rural women’s access to and control over agricultural credit.
	Promote joint land and asset titling between spouses and address the fact that many rural women do not individually own land, which limits their access to credit and rural programs.	Enhance women’s bargaining power and remove land ownership barriers for credit and program access.
	Introduce mandatory gender quotas in rural credit councils and agricultural cooperatives.	Ensure women’s perspectives influence funding and policy decisions.
	Foster gradual, culturally responsive community-level gender awareness initiatives through dialogue and participatory activities, supporting women who wish to explore new opportunities while respecting diverse choices.	Facilitate incremental, community-driven shifts in women’s economic participation and decision-making.
Environmental Health	Link social protection programs with environmental sanitation infrastructure (e.g., improved waste collection systems).	Reduce health risks from improper waste disposal and environmental degradation.
Nutrition and Health	Expand nutritional and hygiene education as part of Bolsa Família conditionalities, delivered through CRAS and primary healthcare teams, with activities directed at the whole household rather than only women.	Encourages shared responsibility for healthy food preparation, reducing the disproportionate domestic burden on women and reinforcing healthier eating habits for all family members.
	Integrate practical meal-planning and healthy cooking workshops into rural extension services, scheduled at times and locations that accommodate women’s agricultural and domestic workload.	Increases feasibility of adopting healthy diets without overburdening women, ensuring interventions are adapted to local rhythms and labor demands

Among the three programmes evaluated, participants highlighted the cistern as most impactful for food production, as home installations saved time previously spent on water collection and enabled cultivation of gardens and animal husbandry.

These findings align with other studies demonstrating that cisterns enhance household food security through reducing time burdens for women and girls, enabling greater participation in education and agriculture [31, 32], and mitigating health risks associated with water collection, which disproportionately affects them in 80% of households lacking on-premises water access [33]. The time savings and health benefits can facilitate a cycle of agricultural improvement, characterized by diversified home gardens, decreased reliance on chemical pesticides, enhanced water quality, and new income streams from surplus vegetable and herb sales [34, 35].

Although adopted by only 5 of the 17 women in the study, the PRONAF also influenced the food production of these participants by enabling their families to buy and sell larger animals, such as goats, sheep, and cattle, and build cisterns. However, decisions on credit usage were made in consultation with their husbands or fathers. This suggests that while women play a crucial role in agricultural food production, their decision-making power is limited to domestic activities, such as managing vegetable gardens and small animals, rather than accessing and utilizing credit.

This study corroborates existing evidence on the topic. In its 2020 evaluation, the Public Policy and Evaluation Council of Brazil (CMAP) reported that women’s access to the PRONAF has not led to productive autonomy or diversification [36]. Arboit et al. [3] concluded that access to credit does not change labor relations or management responsibilities, which remain with the husband. One underlying reason may be women’s limited access to land and other productive assets. Although this study did not directly examine land ownership among participants, national data from the 2017 Brazilian Agricultural Census [37] show that only 19% of rural establishments are headed by women, and these hold fewer productive resources such as machinery, vehicles, or irrigation systems. Limited asset ownership reduces bargaining power and eligibility for credit, as assets are central to women’s ability to negotiate within households and engage in productive decision-making [38]. Studies show that women without land are disadvantaged in both formal and informal credit markets, often needing male guarantors [39], and that even de facto female farmers frequently depend on male cooperation to obtain subsidized loans [40]. Combined with traditional gender norms that confine women to domestic roles and exclude them from financial decisions [41], these barriers help explain PRONAF’s limited contribution to women’s empowerment.

Measures such as joint land and asset titling, and ensuring women’s names appear on livestock and equipment registrations, could directly increase their authority in credit negotiations [38]. Expanding on these points, other recommendations to strengthen rural women’s credit access are offering targeted financial literacy and technical assistance, and introducing mandatory gender quotas in rural credit councils and agricultural cooperatives to ensure women’s priorities are reflected in funding decisions. Internationally, India’s National Rural Livelihood Mission (NRLM) provides a valuable example: it promotes women’s economic autonomy through women-centric Self-Help Groups (SHGs) that foster collective decision-making, enabling access to credit, savings, and capacity-building opportunities [42]. Translating similar measures into practice in Brazil is feasible but requires approaches that engage with the social and cultural realities of rural communities. Gender norms should be addressed through dialogue and participatory activities that build trust [43]. Flexible, culturally sensitive strategies can support women seeking new roles while respecting those who prefer continuity, enabling gradual, community-driven shifts in agricultural decision-making and empowerment.

The analysis of the perceptions of these women regarding food consumption patterns revealed that although the participants emphasized eating fresh or minimally processed foods, they are consuming more ultra-processed products, particularly instant noodles and processed broths. Among all the interviewees, the youngest participant identified pasta as her preferred food, in contrast with other respondents, who consistently cited rice and beans as traditional dietary staples in the region. This singular preference may reflect emerging generational shifts in food habits, potentially indicative of increased exposure to globalized food culture, changing socioeconomic conditions enabling access to prepared foods, or an evolving cultural identity among younger community members [44]. The women interviewed associated the increase in income generated by the BF programme with this transition, suggesting that increased purchasing power alone does not guarantee nutritional enhancement without complementary interventions.

According to IBGE data, ultraprocessed foods continue to displace fresh foods in the Brazilian diet, although the rate of this nutritional transition has slowed from an annual increase of 0.6% points (2002–2009) to 0.3 points (2008–2018) [45]. Quantitative studies conducted by Silvani et al. in 2018 [46] and Almeida et al. in 2020 [47] revealed that although the BF programme increases diversity in the beneficiaries consumption, it does not improve the nutritional quality of food.

Qualitative studies on this topic found that women receiving BF in both urban and rural areas base their food choices on cultural factors and convenience, showing improved food security but no significant nutritional improvement in their diets [48, 49]. Southier et al. reported that rural BF beneficiaries did not improve their diet quality as much as urban beneficiaries did, partly because rural populations have greater access to fresh, home-grown foods and maintain stronger regional dietary customs than ultraprocessed food consumption does [50].

This study aligns with the abovementioned research on the cultural and practical aspects of food consumption while adding new insights into the role of the perceived *status* of certain foods and curiosity about new options, such as Knorr© broth powder. For example, dishes such as pizza were seen as opportunities to eat something different, whereas traditional foods such as *xerém* were associated with times of scarcity and were thus avoided by some participants.

Intersectoral initiatives promoting healthy food options could help reverse this trend. Several scholars suggest that reinforcing Bolsa Família’s conditionalities with nutritional and hygiene education and expanding social assistance reference centers (CRAS) activities could promote healthier eating habits, thereby helping families prioritize more nutritious food choices [49, 51]. Yet, any effective strategy for improving dietary patterns must account for women’s substantial workload and the practical challenges of maintaining healthy meal plans. Our findings reveal that rural women typically bear responsibility for domestic chores, household-adjacent tasks, and agricultural work. In this way, such initiatives can better support rural women when directed at families as a whole, as suggested by the Dietary Guidelines for the Brazilian Population [52], rather than focusing only on women. This approach encourages shared responsibility for healthy food preparation and helps reduce women’s domestic overload. Educational activities also need to consider women’s agricultural and domestic workloads, with schedules and formats adapted to local rhythms and labor demands.

The final dimension examined in this study concerns food waste disposal practices. The participants primarily manage organic waste through animal feeding, effectively minimizing food waste. However, improved access to supermarkets and rising incomes have generated increased waste accumulation, including combustible and noncombustible items such as glass and metal cans. While participants reported burning combustible waste, nonburnable waste presents distinct challenges: glass and metal containers accumulate indefinitely, creating physical hazards and breeding sites for disease vectors when improperly stored.

IBGE data reveal that 60% of rural Brazilian residents continue to burn waste, despite laws assigning waste management to municipalities [53]. This practice directly violates Brazil’s Environmental Crimes Law [54], revealing an implementation gap. Only 6 of the women interviewed believed that a public waste collection system should replace the current method, indicating a lack of awareness of public responsibility for this matter. These findings underscore how social programmes promoting economic development in rural areas must incorporate comprehensive infrastructure to collect waste, considering the toxic emissions from burned waste, environmental degradation from untreated noncombustibles and public health risks from improperly managed waste.

Moreover, the perceived changes in rainfall and agricultural yields align with evidence that climate variability is already affecting rural livelihoods. Adaptive capacity depends on both specific resources (e.g., irrigation) and generic resources (e.g., diversified, climate-neutral income) [55]. While programmes like PRONAF and Cisterns may indirectly strengthen resilience, social protection systems often fall short of addressing long-term climate risks unless they integrate adaptation objectives and ensure equitable access [56]. Although climate change lies beyond the scope of this study, recognising these links points to an important area for future policy and research.

The perceptions narrated by these women show that the cistern programme stands out as uniquely transformative by addressing multiple social dimensions, such as poverty, water insecurity, and agricultural constraints, while respecting cultural foodways. In contrast, although the PRONAF and BF programmes increased access to and diversity in the diets of the participants, they did not address gender disparities in rural areas, indicating missed opportunities for systemic nutritional improvements. As Albuquerque [57] emphasizes, effective food security policies must operationalize multidimensional evaluations that integrate human rights principles and recognize individuals as rights-holders rather than merely policy objects. This requires moving beyond sectoral approaches toward integrated strategies that address the complex, interconnected determinants of rural women’s food practices.

### Strengths and limitations

Importantly, other regional developments, such as housing programmes, improved transportation, increased commerce, a nearby dam, and the rural pension system, may have influenced participants’ food practices, making it difficult to isolate the study’s specific factors. Additionally, the findings rely on participants’ narratives, which may be subject to recall bias. To mitigate this, generalized questions and temporal markers were used in the interviews.

The convenience sampling approach may introduce selection bias, as the sample was restricted to a single community and may not fully represent the broader population [58]. Potential limitations include sampling error due to geographic constraints and motivation bias stemming from participants’ self-selection [59]. Nevertheless, the study’s robustness is strengthened by having interviewed over half of the eligible population in the target community.

The lead researcher’s childhood ties to the region can be a strength of this study, as they may help participants feel more comfortable sharing their experiences with someone familiar with their culture. However, this familiarity might have also influenced their responses. As Mays and Pope (2000) described, reflexivity involves recognizing how both the researcher and the research process shape the data. For this study, the researcher’s background was carefully considered, and personal assumptions and biases were addressed through discussions with the supervisor during the study’s design.

## Conclusion

The perceptions of the women from the Balanço community revealed that the Bolsa Família, Cisterns and PRONAF programmes impacted their food practices primarily by enabling changes in food production, preparation, and consumption. The BF Programme, along with access to water from a cistern, enhanced their ability to grow gardens, raise birds, access and purchase food products, and prepare a wider variety of foods. However, the PRONAF’s impact was minimal due to the limited financial decision-making power of these women and the proper management of the waste generated from these changes is crucial to prevent health issues and environmental damage.

This study underscores the importance of women’s perspectives on the impacts of public policies by giving them a voice in a remote rural area of Northeast Brazil. Understanding their experiences is essential for evaluating these programmes’ effectiveness and developing public policies that promote healthy food practices while addressing the unique challenges women face in rural areas.

## Data Availability

No datasets were generated or analysed during the current study.

## Abbreviations

BF: Bolsa Familia
CMAP: Public Policy and Evaluation Council
CRAS: Social Assistance Reference Center
IBGE: Brazilian Institute of Geography and Statistics
PRONAF: National Programme to Strengthen Family Agriculture
